# TGFβ1 attenuates microglial IL1β release through inhibition of NLRP3 inflammasome priming

**DOI:** 10.3389/fimmu.2025.1623643

**Published:** 2026-01-05

**Authors:** Christopher Kalischer, Phani Sankar Potru, Nele Lehmann, Jannik Jahn, Nikolai Leander Rupp, Natascha Vidovic, Tamara Russ, Susanne Wiemann, Björn Spittau

**Affiliations:** 1 Institute of Anatomy, University of Rostock, Rostock, Germany; 2 Bielefeld University, Medical School OWL, Anatomy and Cell Biology, Bielefeld, Germany

**Keywords:** TGFβ1, microglia, inflammasome, NLRP3, IL1b, LPS

## Abstract

**Introduction:**

Microglia reactivity has been described as a driver of brain tissue damage in multiple neurodegenerative pathologies. One of the key features of reactive microglia is the transcriptional upregulation of in ammatory markers, including components of the NLRP3 inflammasome such as *Nlrp3, Casp1*, and *Il1b*. The NLRP3 inflammasome is a multiprotein complex that plays an important role in several neurodegenerative diseases, being essential for cleavage and subsequent release of IL1b from activated microglia. Transforming growth factor β1 (TGFβ1) is a potent immunoregulatory cytokine with fundamental roles in microglial development, maintenance, and regulation of microglia reactivity.

**Methods:**

Using BV2 cells, primary microglia, qPCR, and western blotting the effect of TGFβ1 on LPS-induced inflammasome priming and activation was addressed. *Cx3cr1CreERT2:R26-YFP: Tgfbr2flox/flox* mice were used to elucidate priming in the absence of microglial TGFβ signalling.

**Results:**

In the present study, we demonstrate that TGFβ1 is able to abrogate LPS-induced transcriptional upregulation of the inflammasome-associated genes *Nlrp3, Casp1*, and *Il1b* in microglia. Moreover, we provide evidence that TGFβ1 attenuates microglial IL1b release after nigericin-triggered NLRP3 inflammasome activation as a consequence of reduced priming.Finally, we demonstrate that silencing of microglial TGFβ signalling *in vivo* results in upregulation of *Casp1, Il18*, and *Il1b*.

**Discussion:**

Together, our data enhance the understanding of how TGFβ1 and microglial TGFβ signaling regulate microglial reactivity, further highlighting the essential functions of TGFβ1 as a potentimmunoregulatory factor for microglia.

## 1 Introduction

Neuroinflammatory reactions in the central nervous system (CNS) have been described in a plethora of pathological conditions and are mainly mediated by microglia, which are the resident immune cells of the CNS (1). Microglia are highly dynamic cells that are able to react to different physiological as well as pathological stimuli by adopting specific reactive states (2). Damage-associated molecular patterns (DAMPs) and pathogen-associated molecular patterns (PAMPs) are involved in microglia reactivity and are recognized by microglial pattern recognition receptors (PRRs) such as Toll-like receptors (TLRs) or NOD-like receptors (NLRs) (3). Among the PRRs triggering microglial reactivity, the intracellular NOD-like receptor protein 3 (NLRP3) has been shown to play essential roles in various neuropathological conditions such as Parkinson´s disease (PD), Alzheimer´s disease (AD), and Amyotrophic lateral sclerosis (ALS) (4–7). NLRP3 contains an N-terminal pyrin domain (PYD) to facilitate interactions with adapter proteins, a nucleotide-binding (NACHT) domain to catalyze adenosine triphosphate (ATP) hydrolysis and to regulate self-oligomerization, as well as a C-terminal leucine-rich repeat (LRR) domain to sense triggers for activation. Upon activation, NLRP3 forms an inflammasome complex including the adaptor protein ASC (apoptosis-associated speck-like protein containing a CARD) and the essential effector protein Caspase-1 (8). Activated Caspase-1 cleaves intracellular interleukin-1β (IL1β) as well as interleukin-18 (IL18) resulting in the secretion of both mature cytokines (9). The canonical NLRP3 inflammasome activation includes a crucial priming step required for the transcriptional upregulation of *Nlrp3*, *Casp1*, *Il1b* as well as *Il18* (10). This priming step has been demonstrated to be initiated by DAMPs and/or PAMPs resulting in subsequent activation of TLRs and NLRs (11). The pleiotropic cytokine transforming growth factor β1 (TGFβ1) plays essential roles during the maturation of postnatal microglia, maintenance of adult homeostatic microglia, and regulation of microglial reactivity under pathological conditions (12). Several studies have demonstrated that TGFβ1 is able to abrogate microglial activation induced by lipopolysaccharide (LPS) and Interferon-γ (IFNγ) by regulating transcriptional upregulation of inflammatory markers (13–15). In the present study, we addressed whether TGFβ1 is able to regulate LPS-driven upregulation of NLRP3 inflammasome components in BV2 cells as well as primary microglia, and subsequently affect the release of IL1β after nigericin-induced inflammasome assembly *in vitro*. We provide evidence that TGFβ1 attenuates microglial IL1β release by inhibiting LPS-triggered transcriptional upregulation of *Nlrp3*, *Casp1*, and *Il1b*. Together, our data further enhance our understanding of how TGFβ1 and microglial TGFβ signaling regulate microglial reactivity.

## 2 Material and methods

### 2.1 Microglia-specific Tgfbr2-knockout mice

Microglia-specific recombination to remove *Tgfbr2* was performed using the inducible Cre-LoxP system. Generation of microglia-specific *Tgfbr2*-knockout mice has been described previously (16). In brief, *Tgfbr2flox/flox* lines were crossed with *Cx3cr1CreERT2* mice. These animals were further crossed with *B6.129×1-Gt(ROSA)26Sortm1(EYFP)Cos/J* mice which carry a stop codon flanked by LoxP before yellow fluorescent protein (YFP) to generate *Cx3cr1CreERT2:R26-YFP: Tgfbr2flox/flox* mice. The mice were maintained at a temperature of 22 ± 2 °C, following a 12-h light/dark cycle, with unlimited access to chow and water. All animal experiments were performed in strict adherence with the German Federal and local ethical guidelines (2024-248-Grundantrag). To induce the recombination, adult male and female mutant mice were fed with chow containing tamoxifen citrate (TAM chow) (500mg/kg body weight, ssniff, Soest, Germany) and/or control chow (ssniff, Soest, Germany) for 4 weeks. The mice were then euthanized and brains were promptly removed. Microglia were then isolated using the Adult Brain Dissociation Kit (130-107-677, Miltenyi Biotec, Bergisch Gladbach, Germany) and MACS technology as per the manufacturer´s instructions. Next, magnetic labelling and flow cytometric analysis of microglia were performed. Briefly, microglia were incubated with CD11b MicroBeads (130-126-725, Miltenyi Biotec, Bergisch Gladbach, Germany) and subsequently separated using MS Columns (130-042-201, Miltenyi Biotec, Bergisch Gladbach, Germany) yielding CD11b enriched cell population. Flow cytometry was performed using *u*sing MACSQuant 10 and MACSQuantify Software V2.13.3. Cells were stained with anti-mouse CD11b-Vioblue (130-113-810, Miltenyi Biotec, Bergisch Gladbach, Germany) at a concentration of 1:50 in flow cytometry buffer for 10 min. Afterwards, cells were washed and applied to the flow cytometer. First, cells were gated as single events and then for YFP^+^ events as the cells that underwent recombination express YFP due to the deletion of preceding stop codon. The separated cells were then subjected to RNA isolation and qPCR analysis as described in the RNA isolation and quantitative RT-PCR sections.

### 2.2 Primary microglia cultures

Primary microglia (pMG) were prepared and cultured as previously described (17). Vessels and meninges were removed from the brains of P0/P1 C57BL/6JRj mice (Janvier), and brains were washed and collected in ice-cold Hank´s Buffered Salt Solution (HBSS, Gibco, Germany). After enzymatic dissociation with Trypsin-EDTA (Gibco, Germany) for 15 minutes at 37 °C, an equal volume of fetal calf serum (FCS, Gibco, Germany) and DNase (Roche, Mannheim, Germany) at a final concentration of 0.05 mg/ml was added. Cells were dissociated using wide- and narrow-bored polished Pasteur pipettes and further centrifuged and resuspended in DMEM/F12 medium (Gibco, Germany) containing 10% FCS and 1% penicillin/streptomycin (Invitrogen). Dissociated cells from 2–3 brains were plated on poly-D-lysine-coated (Sigma-Aldrich, Schnelldorf, Germany) 75 cm^2^ culture flasks. Cells were kept in a 5% CO_2_/95% humidified atmosphere at 37 °C. After 10–14 days in culture, microglia were shaken off (250–300 rpm for 1 h) from adherent astrocytes and plated according to the experimental designs. Treatment with recombinant human TGFβ1 (Peprotech, Hamburg, Germany) was performed at a concentration of 5 ng/mL while LPS (L8274, Sigma-Aldrich, Schnelldorf, Germany) was added at a concentration of 1 µg/mL. For the activation of the inflammasome, Nigericin (InvivoGen, Toulouse, France) was used at a concentration of 1.34 mM. For *in vitro* TGFβ signaling inhibition, primary microglia were treated with SB431542 (Selleckchem, 5µM) for 24h or left untreated. All the treatments were performed under serum-free conditions.

### 2.3 BV2 cell culture

BV2 cells were cultured as described by Zhou et al. (8). Cells were kept in DMEM/F12 culture medium (ThermoFisher Scientific) supplemented with 10% fetal calf serum (FCS) and 1% penicillin/streptomycin (Invitrogen) and maintained at 37 °C in a 5% CO_2_/95% humidified atmosphere. Treatments with 1 µg/ml LPS and/or 5 ng/ml TGFβ1 (Peprotech, Hamburg, Germany) for qPCR and western blotting experiments were always performed under serum-free conditions.

### 2.4 RNA isolation and reverse transcription

RNA was isolated from pMG using TRIzol reagent (Invitrogen, Karlsruhe, Germany) according to manufacturer´s instructions. RNA concentration and quality were determined using the NanoDrop 2000 (Thermo Scientific, Germany). 1 µg total RNA from each sample was reverse transcribed to cDNA using Protoscript^®^ II First Strand cDNA Synthesis Kit (#E6560S, New England Biolabs, Frankfurt, Germany) according to the manufacturer’s instructions.

### 2.5 Quantitative RT-PCR

Quantitative RT-PCR was performed using the CFX Connect™ System (Bio-Rad, München, Germany) in combination with the SYBR Green GoTaq^®^ qPCR Kit (A6002, Promega, Madison, WI, USA). 5 µl of cDNA template was used in 20 µl reaction mixture. Results were analyzed using the CFX Connect™ System (Bio-Rad, München, Germany) Software and the comparative CT method. All data are expressed as 2^-ΔΔCT^ for the gene of interest normalized to the housekeeping gene Gapdh and presented as fold change relative to controls. The following primers have been used throughout this study: Nlrp3*for* 5´-CACCTTGTGGAGTACATGGAAC-3´, Nlrp3*rev* 5´- CTACCTCCCTTTCAAGACGGT-3´ [NM_133859.2], Casp1*for* 5´-CACCTTGTGGAGTACATGGAAC-3´, Casp1*rev* 5´- CTACCTCCCTTTCAAGACGGT-3´ [NM_133859.2], Il18*for* 5´-CACCTTGTGGAGTACATGGAAC-3´, Il18*rev* 5´- CTACCTCCCTTTCAAGACGGT-3´ [NM_133859.2], Il1b*for* 5´-CACCTTGTGGAGTACATGGAAC-3´, Il1b*rev* 5´- CTACCTCCCTTTCAAGACGGT-3´ [NM_133859.2], Gapdh*for* 5´-GGCATTGCTCTCAATGACAA-3´, Gapdh*rev* 5´- ATGTAGGCCATGAGGTCCAC-3´ [NM_001289726].

### 2.6 Protein isolation and western blotting

Total proteins were extracted from primary microglia with RIPA Buffer (#89900, Thermo Fisher Scientific, Germany) according to the manufacturer’s instructions. Proteins were also isolated from conditioned cell culture medium. Briefly, acetone was added to culture medium at a ratio of 3:1 and incubated at -20 °C for 30min. Then the mixtures were centrifuged at maximum speed for 20min. The supernatant was discarded and the resultant pellet was resuspended in 1X PBS. Protein concentrations for both cell lysates and supernatants were determined using Pierce™ BCA Protein Assay Kit (#23225, Thermo Fischer Scientific, Germany) as per the manufacturer’s instructions. 10 mg of protein per lane was loaded into 4–20% Mini-PROTEAN^®^ TGX™ Precast Gels (Bio-Rad, Munich, Germany). Electrophoresis was performed at 120 volts for 90 minutes. Then, the proteins were transferred using the Trans-Blot^®^Turbo™ RTA Midi PVDF Transfer Kit (#1704275, Bio-Rad, Munich, Germany) for the Trans-Blot^®^Turbo™ Transfer System (Bio-Rad, Munich, Germany) onto a PVDF membrane. The membranes were then briefly rinsed with 1X Tris-buffered saline with 0.1% Tween^®^ 20(1X-TBST) and blocked with 5% bovine serum albumin (Carl Roth, Karlsruhe, Germany) in 1X-TBST for 90 mins at room temperature. They were later incubated with primary antibodies against Il1β (AF-401-NA, R&D Systems, 1:500), Nlrp3 (AG-20B-0014, Adipogen, 1:500), Caspase-1 (AG-20B-0042, Adipogen, 1:500) and β-Actin (#4967, Cell Signaling,1:2000) overnight at 4 °C. After the primary antibody incubation, membranes were washed with 1X-TBST three times for 10 minutes at room temperature. Then, the membranes were incubated with horseradish peroxidase (HRP)-conjugated anti-goat (sc-2354, Santa Cruz Biotechnology, 1:2000), anti-rabbit (#7074, Cell Signaling, 1:2000) and anti-mouse (#7076, Cell Signaling, 1:2000) in 1X-TBST at room temperature for 90 minutes. Then, the proteins were detected using SignalFire™ Elite ECL Reagent (#12757, Cell Signaling) and visualized using the ChemiDoc MP imaging system (Bio-Rad, Munich, Germany). Densitometric analysis of protein bands was carried out using ImageJ software (National Institutes of Health, Bethesda, MD, United States).

### 2.7 ILβ1 ELISA

ILβ1 was detected in in medium from pMG cultures treated for 6 h followed by additional 2 h of nigericin treatment using a mouse IL-1 beta/IL-1F2 ELISA Kit (#MLB00C, R&D Systems, Wiesbaden-Nordenstedt, Germany) according to the manufacturer´s instructions. Absorbances were detected using a Multiskan FC plate reader (Thermo Fischer) and concentrations of ILβ1 were calculated from standard curves using GraphPad Prism10 software (GraphPad Software Inc., La Jolla, CA, USA).

### 2.8 Data availability and analysis

The micro-array data for pMG treated with TGFβ1 used in the study is available on the Gene Expression Omnibus database (GEO) under the number GSE115652. Heatmap was made using SRplot (18). Pathway enrichment analysis was performed using Metascape (19).

### 2.9 Statistics

Data are given as means ± standard error of the mean (SEM). Statistical differences between two groups were determined using Student´s *t*-test. Multiple-group analysis was performed using one-way ANOVA followed by Bonferroni´s multiple comparison post-test. *P*-values ≤ 0.05 were considered as being statistically significant. All statistical analyses were performed using the GraphPad Prism10 software (GraphPad Software Inc., La Jolla, CA, USA).

## 3 Results

### 3.1 Transcriptomic analysis reveals TGFβ1-regulated expression of inflammasome genes in primary microglia

**Figure 1:**
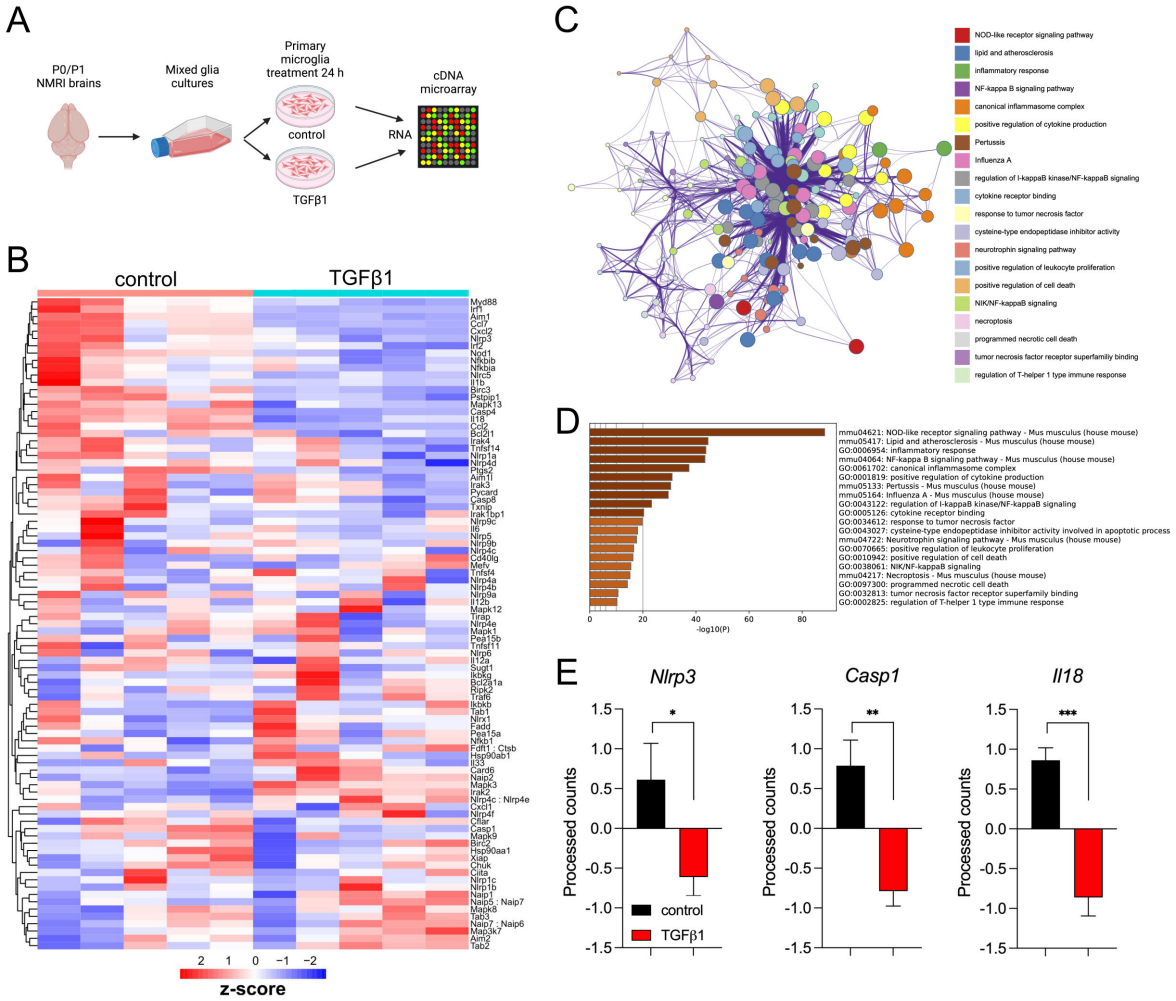
Transcriptomic profiling reveals TGFβ1-regulated inflammasome-related genes in primary microglia. Schematic representation of the cDNA microarray experiment of primary microglia treated with TGFβ1 for 24 hours. Created with BioRender.com**(A)**. Heatmap of inflammasome-related genes. The color key corresponds to the row Z-score employed to identify gene expression patterns **(B)**. Enriched terms colored by cluster ID. A circular node represents each term, and the size of the node corresponds to the number of genes that are implicated in each term from the input list, while the color represents the term cluster identity. Nodes sharing the same cluster occur in proximity. The most significant terms from each cluster are described in the label on the right **(C)**. Functional enrichment heatmap of the gene list showing the top 20 enriched terms colored by *p*-values. The darker the color, the lower the p-value **(D)**. Bar graphs representing expression patterns of important inflammasome-related genes **(E)**.

Inflammasome-relevant genes were screened from a previously published study (20). To confirm the pathways represented by the gene set from Hytti et al., 2021, pathway enrichment analysis was performed for GO Biological Processes, GO Cellular Components, GO Molecular Functions, and KEGG Pathway using Metascape. All genes in the genome have been used as the enrichment background. This analysis has revealed that pathways such as the NOD-like receptor signaling pathway, NF-κB signaling pathway, canonical inflammasome assembly, and cytokine-producing pathways are highly enriched (**Figures 1C, D**). In order to analyze the effect of TGFβ1 signaling on inflammasome-related genes, a previously published transcriptomic analysis (16) in which pMG cultures were treated with 5 ng/ml TGFβ1 for 24 h was used (**Figure 1A**). Results from this analysis have shown that TGFβ1 treatment reduces the expression of inflammasome-related genes such as *Nlrp3, Nlrlp1a, Casp1, Casp4*, and *Aim1* (**Figures 1B, E**). In addition to this, genes involved in several other inflammatory pathways were also downregulated. Toll-like receptor 2 (TLR2) pathway genes such as *Myd88* and *Irak4*, Nuclear factor-κB (NF-κB) signaling genes like *Nfkbia, Nfkbib*, and *Bcl2l1*, interferon regulatory factor (IRF) family genes *Irf1* and *Irf2*, and *Il1b* and *Il18* belonging to interleukin (IL) receptor signaling were among the genes downregulated upon TGFβ1 treatment (**Figures 1B, E**). Overall, these findings indicate that TGFβ1 has a significant regulatory effect on the genes involved in the inflammasome and other inflammatory pathways by significantly reducing priming of primary microglia.

### 3.2 TGFβ1 inhibits LPS-induced upregulation of NLRP3 inflammasome genes in BV2 cells

**Figure 2:**
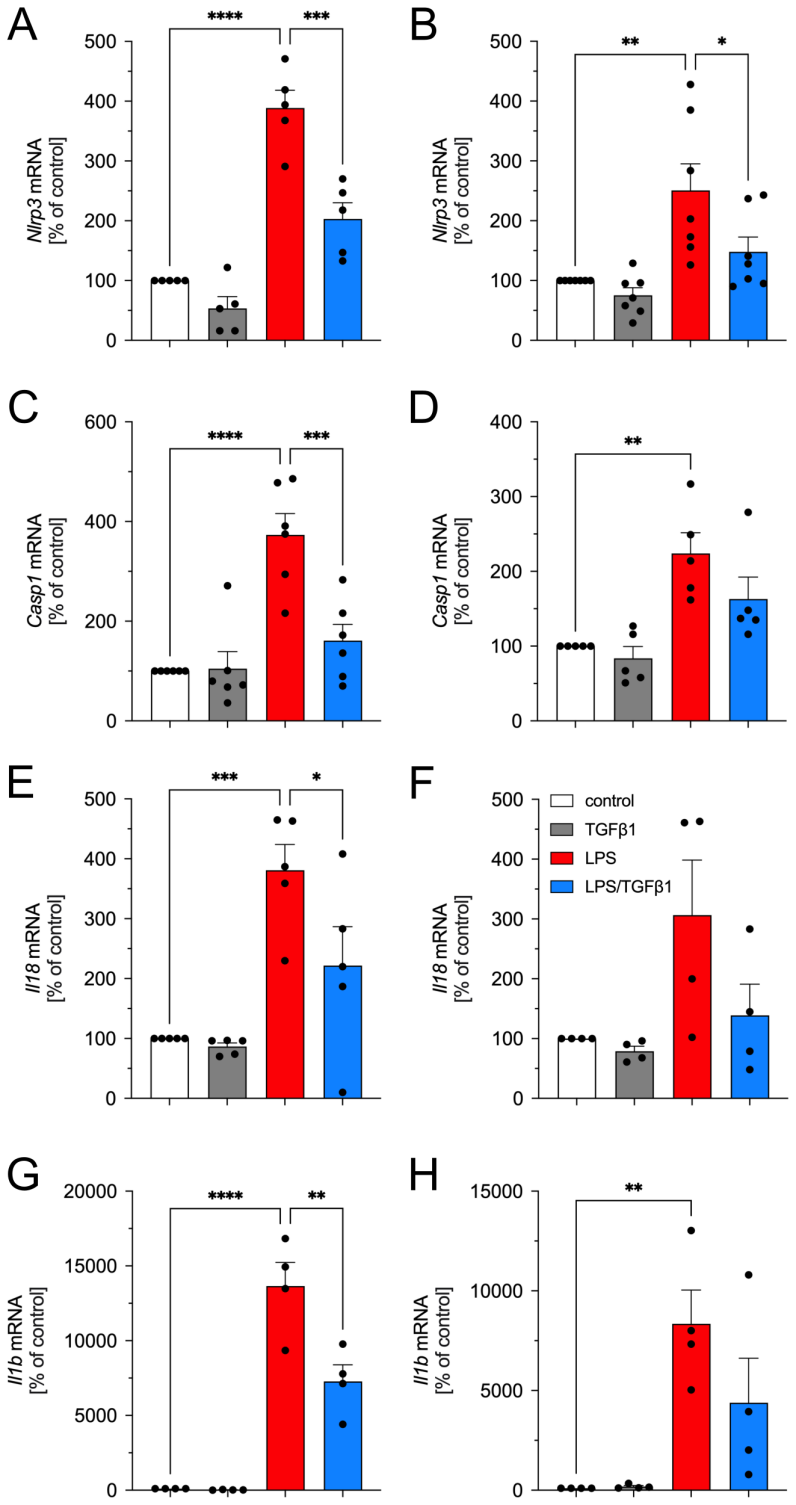
TGFβ1 inhibits LPS-induced transcriptional upregulation of *Nlrp3*, *Casp1*, *Il18*, and *Il1b* in BV2 cells. Expression of *Nlrp3***(A, B)**, *Casp1***(C, D)**, *Il18***(E, F)**, and *Il1b***(G, H)** was analyzed after treatment with TGFβ1 (5 ng/ml), LPS (1 µg/ml) or the combination of both factors for 6 h **(A, C, E, G)** and 12 h **(B, D, F, H)** using qPCR. Data are given as means ± SEM from at least three independent experiments. P-values derived from one-way ANOVA followed by Tukey´s multiple comparison tests are *p < 0.05, **p < 0.01, ***p < 0.001, and ****p < 0.0001.

**Figure 3:**
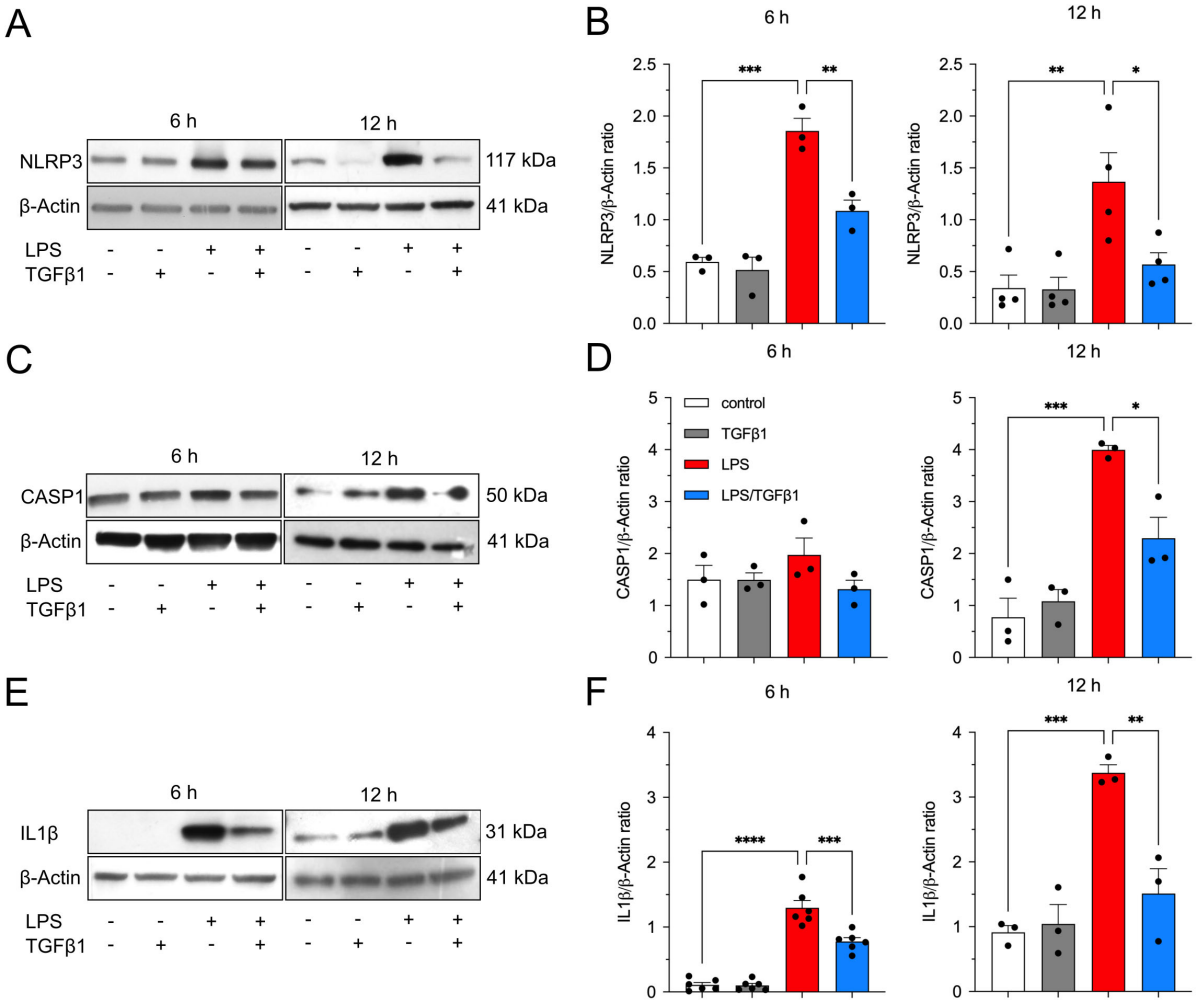
TGFβ1 significantly reduces LPS-induced increases in NLRP3, CASP1, and IL1β protein levels in BV2 cells. After treatment with TGFβ1 (5 ng/ml), LPS (1 µg/ml) or the combination of both factors for 6 h and 12 h, protein levels were analyzed using western blotting. Representative western blot images for both experimental time points are depicted for NLRP3 **(A)**, CASP1 **(C)**, and IL1 β **(E)**. Quantifications after normalization using β-Actin are presented for NLRP3 **(B)**, CASP1 **(D)**, and IL1β **(F)**. Data are given as means ± SEM from at least three independent experiments. P-values derived from one-way ANOVA followed by Tukey´s multiple comparison tests are *p < 0.05, **p < 0.01, ***p < 0.001, and ****p < 0.0001.

In the next step, the microglia cell line BV2 was used to evaluate the effects of TGFβ1 on the expression of NLRP3 inflammasome-related genes. As shown in **Figure 2**, treatment of BV2 cells with LPS at a concentration of 1 µg/ml for 6 h resulted in significant upregulation of *Nlrp3* (**Figure 2A**), *Casp1* (**Figure 2C**) as well as the cytokines *Il18* (**Figure 2E**) and *Il1b* (**Figure 2G**). Whereas the treatment with TGFβ1 (5 ng/ml) had no significant effects on the expression of *Nlrp3*, *Casp1*, *Il18*, and *Il1b* after 6 h, co-treatment with LPS and TGFβ1 resulted in significantly reduced expression of *Nlrp3*, *Casp1*, *Il18*, and *Il1b* as compared to the treatment with LPS alone (**Figures 2A, C, E, G**). Similar results were obtained after treatment of BV2 cells for 12 h. Increased expression of *Nlrp3*, *Casp1*, and *Il1b* were detected after treatment with LPS alone (**Figures 2B, D, H**). Although LPS treatment resulted in higher *Il18* expression, the increase was found not to be significant (**Figure 2F**). The incubation of BV2 cells with a combination of LPS and TGFβ1 resulted in decreased expression of *Nlrp3*, *Casp1*, *Il18*, and *Il1b* after 12h when compared to LPS treatments alone (**Figures 2B, D, F, H**). However, TGFβ1 only significantly inhibited LPS-induced upregulation of *Nlrp3* after treatment for 12 h (**Figure 2B**). Taken together, these data indicate that TGFβ1 can abrogate LPS-induced transcriptional upregulation of the inflammasome-related genes *Nlrp3*, *Casp1*, *Il18*, and *Il1b* in BV2 cells. We next analyzed the protein levels of NLRP3, CASP1, and IL1β after treatment of BV2 cells for 6 h and 12 h using western blots. As shown in **Figure 3A**, LPS treatment alone resulted in a significant increase of NLRP3 after 6h and 12 h. Quantification of NLRP3 confirmed this observation (**Figure 3B**). TGFβ1 was able to significantly inhibit the LPS-induced increase of NLRP3 at both time points (**Figures 3A, B**). Analysis of CASP1 protein levels revealed a slight increase after LPS treatment for 6 h and a significant increase after 12 h (**Figures 3C, D**). Again, TGFβ1 significantly blocked the LPS-induced increase of CASP1 levels after treatment for 12 h (**Figures 3C, D**). Finally, intracellular levels of IL1β were highly elevated after treatment of BV2 cells with LPS for 6 h as well as 12 h (**Figures 3E, F**). At both time points analyzed, TGFβ1 was able to significantly inhibit the LPS-triggered increase of IL1β. In accordance with the qPCR data, TGFβ1 inhibited the LPS-mediated increases of NLRP3, CASP1, and IL1β in the microglia cell line BV2.

### 3.3 TGFβ1 inhibits LPS-induced upregulation of NLRP3 inflammasome genes in primary microglia

**Figure 4:**
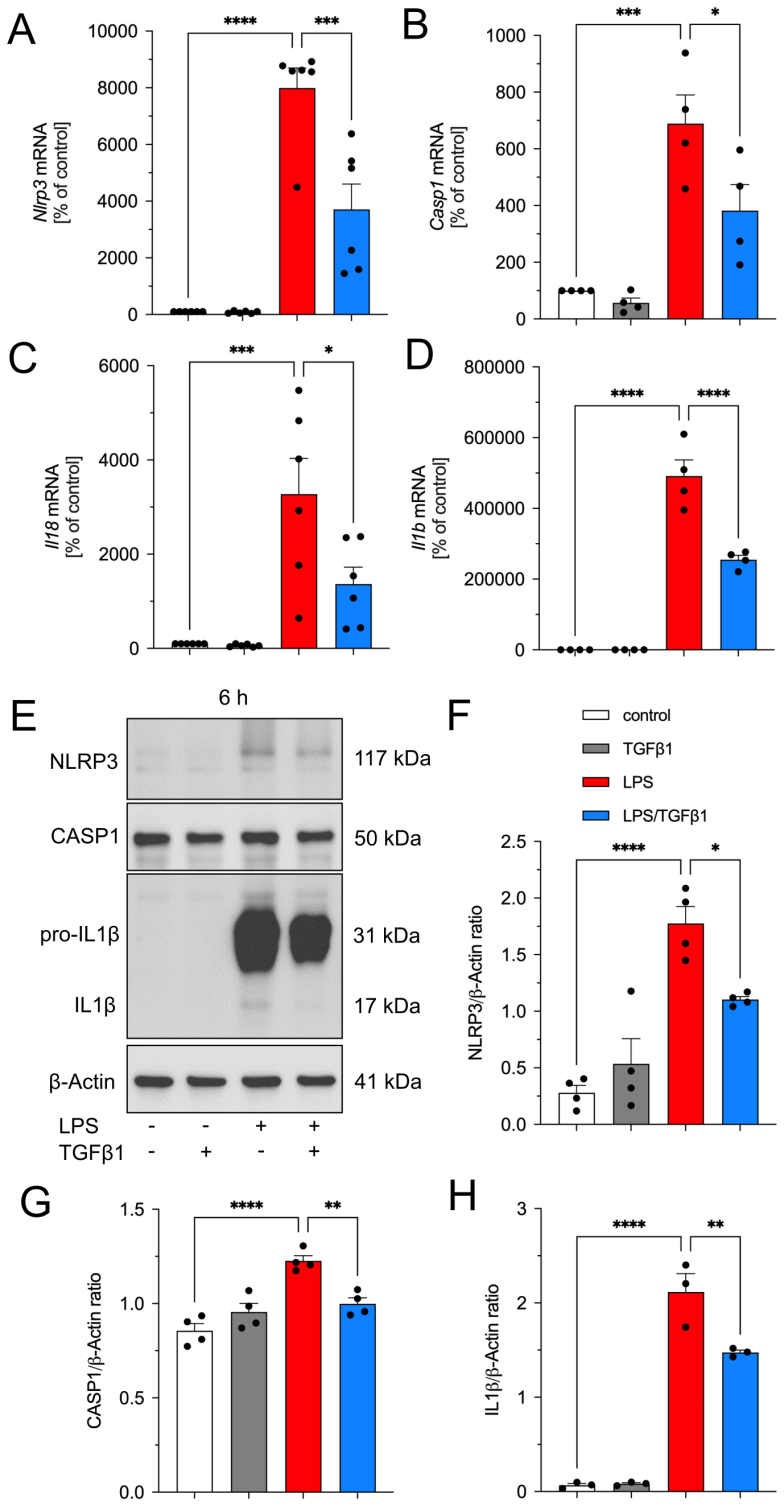
TGFβ1 inhibits LPS-induced expression of *Nlrp3*, *Casp1*, *Il18*, and *Il1b* in primary microglia. Microglia were treated with TGFβ1 (5 ng/ml), LPS (1 µg/ml) or the combination of both factors for 6 h and RNA and total proteins were isolated. TGFβ1 significantly inhibits LPS-induced upregulation of *Nlrp3***(B)**, *Casp1***(B)**, *Il18***(C)**, and *Il1b***(D)** in primary microglia. TGFβ1 abrogates LPS-induced increases in NLRP3, CASP1, and IL1β protein levels in microglia. Representative western blot images are depicted **(E)**. Quantifications after normalization using β-Actin are presented for NLRP3 **(F)**, CASP1 **(G)**, and IL1β **(H)**. Data are given as means ± SEM from at least three independent experiments. P-values derived from one-way ANOVA followed by Tukey´s multiple comparison tests are *p < 0.05, **p < 0.01, ***p < 0.001, and ****p < 0.0001.

Since it has been reported that BV2 display a distinct and different gene expression profile compared to pMG, we aimed to confirm the abovementioned results using primary mouse microglia cultures. Due to the limited availability of pMG, we decided to focus on treatments for 6 h. Moreover, this timepoint has been used in several studies addressing the expression of inflammasome-related genes as well as inflammasome assembly in pMG (21, 22). As demonstrated in **Figure 4**, treatment of pMG with LPS (1 µg/ml) resulted in robust transcriptional upregulation of *Nlrp3* (**Figure 4A**), *Casp1* (**Figure 4B**), *Il18* (**Figure 4C**), as well as *Il1b* (**Figure 4D**). Noteworthy, pMG reacted with stronger increase of all 4 genes analyzed when compared to BV2 cells. Similar to the observed effects of TGFβ1 in BV2 cells, LPS-induced upregulations of *Nlrp3*, *Casp1*, *Il18*, and *Il1b* were significantly abrogated after co-treatment with TGFβ1 (**Figures 4A–D**). Analysis of NLRP3, CASP1, and IL1β protein levels revealed significant increases after treatment with LPS for 6 h (**Figures 4E–H**). In accordance with the transcriptional data, TGFβ1 significantly inhibited LPS-induced effects on NLRP3, CASP1 as well as IL1β in pMG (**Figures 4E–H**). It should be mentioned that the abundancy of pro-IL1β (31 kDa) after LPS treatment was much stronger than in BV2 cells indicating a more robust reaction of pMG to LPS treatment. Moreover, we were also able to detect low amounts of mature IL1β at a molecular weight of 17 kDa after LPS treatment. Together, these data confirm our result from BV2 cells and further demonstrate that TGFβ1 can inhibit LPS-induced priming as evidenced by abrogated upregulation of inflammasome-related genes in pMG.

### 3.4 TGFβ1 reduces IL1β secretion after Nigericin-induced NLRP3 inflammasome assembly

**Figure 5:**
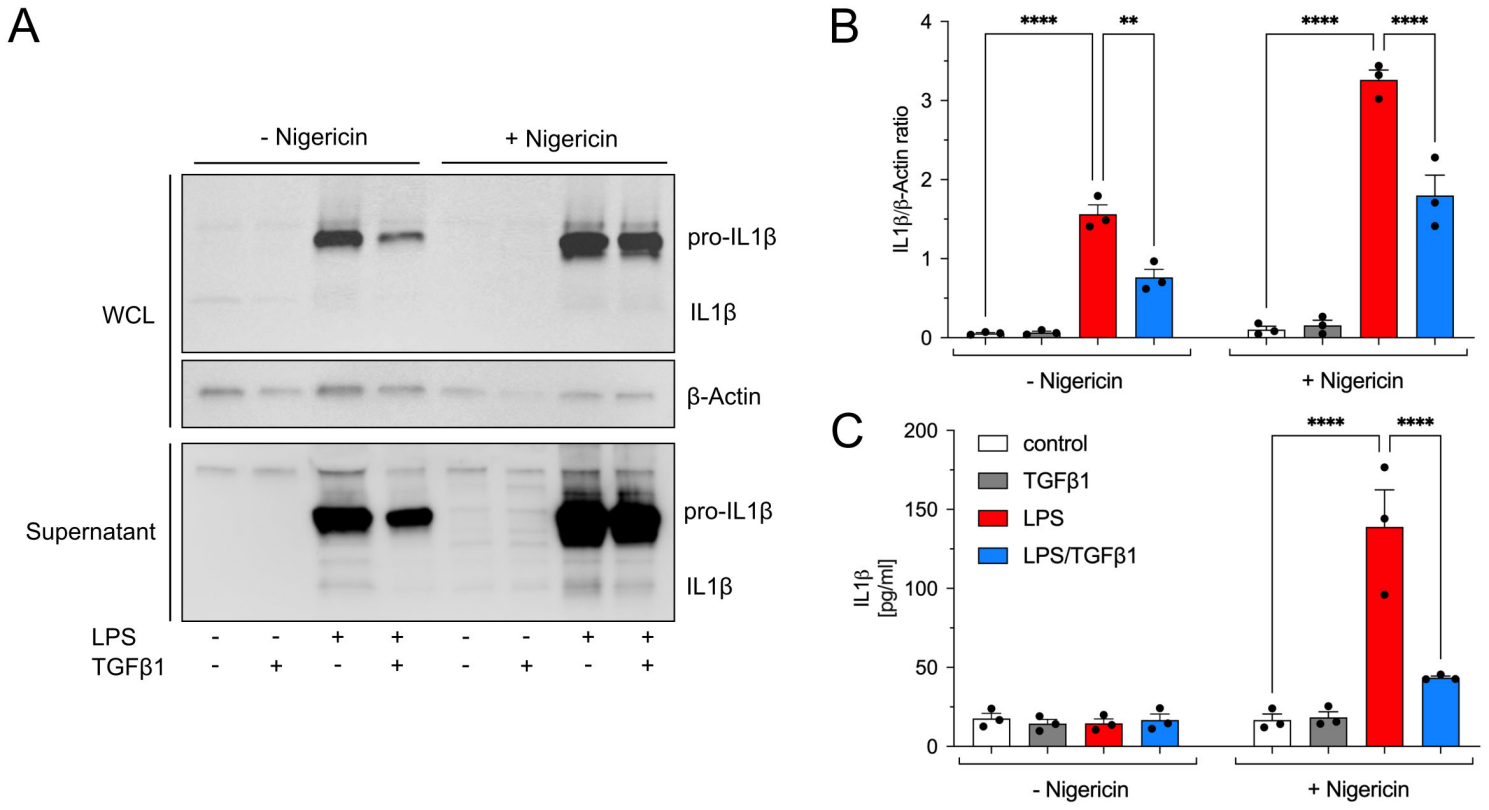
TGFβ1 reduces release of inflammasome cleaved IL1β from primary microglia. Primary microglia were treated with TGFβ1 (5 ng/ml), LPS (1 µg/ml) or the combination of both factors for 6 h and inflammasome assembly was triggered using Nigericin (1.34mM/ul) treatment for additional 2 h. Proteins were isolated from whole cell lysates (WCL) and supernatants and pro- IL1β and cleaved IL1β was visualized. Representative western blot images are depicted **(A)**. Quantifications of pro-IL1β after normalization using β-Actin is presented **(B)**. Total levels of cleaved and bioactive IL1β in supernatants were quantified using ELISA. Quantifications of released IL1β are given **(C)**. Data are given as means ± SEM from at least three independent experiments. P-values derived from one-way ANOVA followed by Tukey´s multiple comparison tests are **p < 0.01, and ****p < 0.0001.

In the next step, we analyzed inflammasome assembly and subsequent secretion of mature IL1β from pMG using Nigericin, a well-established inflammasome activator. Therefore, pMG were treated with LPS, TGFβ1 as well as the combination of both factors for 6 h. Cells were further treated for 2 h with or without Nigericin (1.34 mM). Whole cell lysates (WCL) and supernatants were used for western blots as well as IL1β ELISA. As shown in **Figure 5A**, Nigericin treatment resulted in robust release of pro-IL1β and mature IL1β from pMG. In the presence of TGFβ, Nigericin-triggered secretion of IL1β was significantly reduced (**Figures 5A, B**). Using an ELISA, concentrations of secreted IL1β after different treatments with and without Nigericin-induced inflammasome activation were analyzed in microglia supernatants. As shown in **Figure 5C**, very low levels of IL1β were detectable in the absence of Nigericin. However, Nigericin-triggered inflammasome activation resulted in significant secretion of IL1β (138.9 ± 23.44 pg/ml) in LPS-treated pMG. Co-treatment with TGFβ1 significantly reduced the levels of secreted IL1β (43.6 ± 0.94 pg/ml). These TGFβ1 effects are comparable to the total protein analyses shown in **Figure 4**, indicating that TGFβ1 is able to inhibit LPS-induced upregulation of inflammasome-related genes but seems to have a minor influence on Nigericin-induced inflammasome assembly and activation. In summary, our data demonstrate that TGFβ1 negatively regulates LPS-induced upregulation of the inflammasome genes *Nlrp3*, *Casp1*, and *Il1b* resulting in reduced secretion of mature IL1β from pMG after inflammasome activation.

### 3.5 Silencing of microglial TGFβ signaling increases expression of inflammasome genes

**Figure 6:**
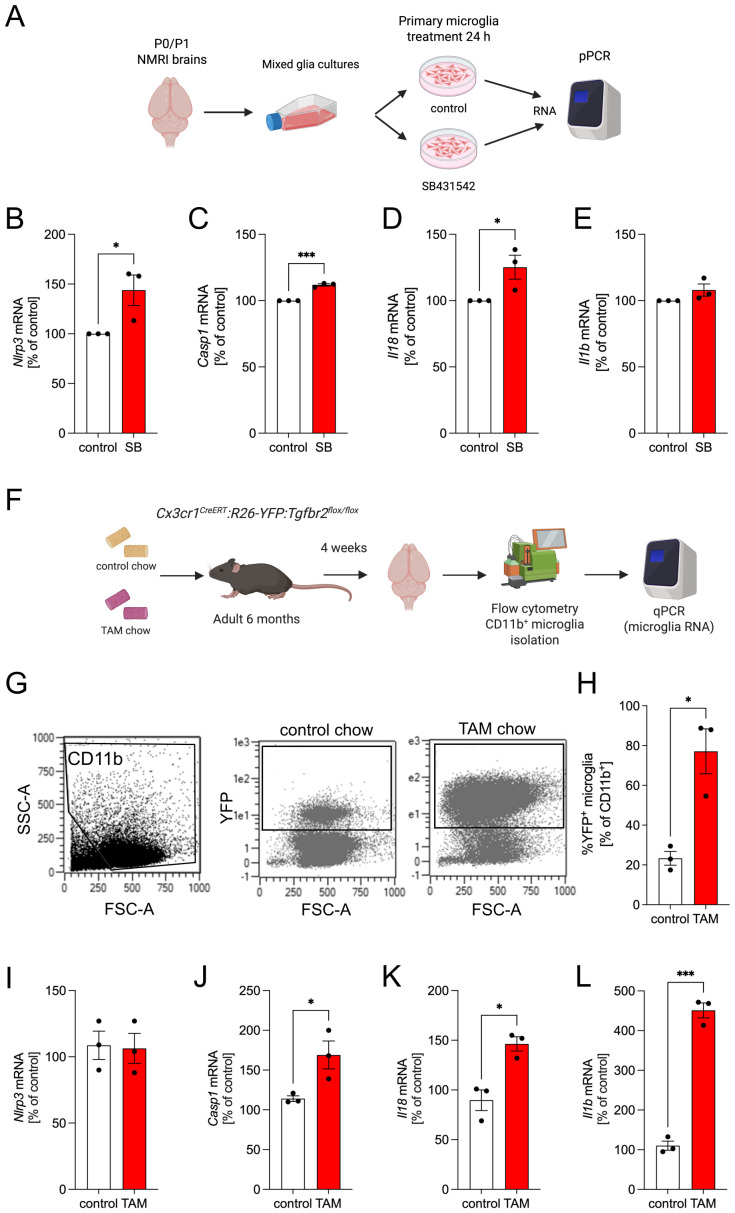
Silencing of microglial TGFβ signalling leads to increased expression of inflammasome genes. Schematic workflow for TGFβ signalling inhibition *in vitro***(A)**. Expression of *Nlrp3***(B)**, *Casp1***(C)**, *Il18***(D)**, and *Il1b***(E)** was analyzed after treatment with SB431542 or the solvent control for 24 h. Data are given as means ± SEM from three independent experiments. P-values derived from paired student´s t-test are *p < 0.05 and ***p < 0.001. Schematic workflow for microglia-specific deletion of Tgfbr2 in *Cx3cr1CreERT2:R26-YFP: Tgfbr2flox/flox* mice *in vitro***(F)**. Representative flow cytometry plots showing CD11b^+^ microglia after immunomagnetic separation and YFP^+^ microglia from control chow and TAM chow mice **(G)**. Quantification of YFP^+^ microglia after flow cytometry. Data are given as means ± SEM from three animals per group **(H)**. Expression of *Nlrp3***(I)**, *Casp1***(J)**, *Il18***(K)**, and *Il1b***(L)** was analyzed using qPCR after 4 weeks of tamoxifen-induced silencing of microglial TGFβ signalling. Data are given as means ± SEM from three animals per group. P-values derived from unpaired student´s t-test are *p < 0.05 and ***p < 0.001.

In order to check whether inhibition of microglial TGFβ signalling affects the transcription of the inflammasome genes *Nlrp3*, *Casp1*, *Il18*, and *Il1b*, pMG were treated with SB431542, a potent inhibitor TGFβ type I receptor ALK5, for 24 h or left untreated (**Figure 6A**). As shown in **Figures 6B–D**, qPCR analysis revealed that inhibition of TGFβ signaling resulted in significantly increased expression of *Nlrp3*, *Casp1*, and *Il18* in primary microglia. However, expression levels of *Il1b* were only slightly increased without reaching significancy (**Figure 6E**). These findings indicate that TGFβ signalling in microglia controls the expression levels of inflammasome genes. Since serum-free cultures of primary microglia cannot be treated for long time periods and hardly resemble the situation *in vivo*, we further used microglia-specific *Tgfbr2* mutant mice to address whether lack of TGFβ signaling affects expression of inflammasome genes. We have previously shown that targeting *Tgfbr2* in microglia leads to impaired homeostasis and render them immunologically primed (16). In order to test if this priming comprises of inflammasome related gene activation, *Cx3cr1CreERT2:R26-YFP: Tgfbr2flox/flox* mice were fed with control chow or chow containing tamoxifen (TAM) for 4 weeks were analysed in this study (**Figure 6F**). Initially, flow cytometry analysis showed increased YFP expression in TAM chow mice when compared to mice that received control chow, thereby confirming efficient recombination and deletion of *Tgfbr2* in microglia (**Figures 6G, H**). Subsequent qPCR analysis of RNA isolated from microglia after CD11b-based immunomagnetic cell separation revealed that deletion of microglial *Tgfbr2* results in significantly increased expression of *Casp1, Il18*, and *Il1b* (**Figures 6J–L**), while *Nlrp3* transcriptional levels were comparable between both groups (**Figure 6I**). Together, these results indicate that silencing of microglial TGFβ signalling leads to increased expression of inflammasome genes, a change that could be an important feature of immunologically primed *Tgfbr2*-deficient microglia.

## 4 Discussion

Microglia-mediated neuroinflammation is a key component of various neurodegenerative diseases, including Alzheimer’s disease (AD), Parkinson’s disease (PD), and multiple sclerosis (MS) (23). Chronic microglial activation associated with these conditions aggravates neuronal damage by maintaining a pro-inflammatory environment, in which the NLRP3 inflammasome plays a central role. This multiprotein complex regulates the maturation and secretion of pro-inflammatory cytokines such as IL1β and IL18, contributing to neurodegeneration (24, 25). Here, we demonstrate a regulatory role for TGFβ1 in suppressing inflammasome-related gene expression in both pMG and BV2 microglial cells. Furthermore, we show that TGFβ1 reduces the LPS-induced IL1β secretion following Nigericin-triggered inflammasome activation. Moreover, we demonstrate that silencing microglial TGFβ signalling by deletion of *Tgfbr2* results in upregulation of inflammasome genes. These findings position TGFβ1 as a potent modulator of microglial priming, subsequently controlling inflammasome activity with the ability to ease neuroinflammatory processes during the course of neurodegeneration.

TGFβ1 has been shown to be important in establishing the microglia-specific homeostatic marker expression during early postnatal development and maintaining it during adulthood (26). Disruption of TGFβ signaling by targeting microglia-specific *Tgfbr2* was shown to render them immunologically primed (16) as well as inducing region-specific pathology in the CNS (27). Moreover, several studies have shown that microglial TGFβ1 plays a critical role in suppressing a shift towards a pro-inflammatory phenotype in response to disease and toxin exposure. One mechanism through which TGFβ1 signaling can accomplish this is by reducing lipid droplet accumulation, as shown by recent studies (28, 29). Therefore, to investigate whether TGFβ1 signaling sustains microglial homeostatic phenotype through inhibition of inflammasome-related genes, we analyzed expression patterns of genes identified by Hytti et al. (20) within a publicly available transcriptomic dataset of TGFβ1-treated microglia by Zöller et al. (16). The transcriptomic analysis identified significant downregulation of inflammasome-related genes, including *Nlrp3*, *Casp1*, and *Il18* (**Figures 1B, E**). In contrast, increased levels of *Nlrp3* and Casp*1* were previously shown in amyloid-beta (Aβ) treated BV2 microglia cells, and in the cortex and hippocampal tissues of the APP/PS1 AD mouse model. Moreover, significantly higher levels of IL1β and IL-18 were found in the supernatant of BV2 cell cultures and brain tissues suggesting an elevated release of these cytokines. These findings confirm the activation of the inflammasome under neurodegenerative conditions (30). Taken together, a striking contrast in the expression patterns of inflammasome-related genes in response to TGFβ1 and Aβ treated microglia aligns with prior studies emphasizing the role of TGFβ1 in maintaining microglia quiescence through repressing pro-inflammatory pathways (17). Our work extends this understanding and positions the NLRP3 inflammasome as a potential target for TGFβ signaling mediated anti-inflammatory effects.

Numerous studies have consistently demonstrated heightened NLRP3 inflammasome and CASP1 activity across diverse cell types and in a wide range of inflammatory conditions (6, 31–34). Our data also show increased NLRP3 and CASP1 protein levels upon LPS treatment in both BV2 cells and pMG, while co-treatment with TGFβ1 reduced LPS-induced upregulation. Interestingly, previous studies have shown similar effects on NLRP3 components by TGFβ1. In an LPC-induced demyelinating mouse model, TGFβ1 administration was shown to effectively ameliorate proinflammatory microglia pyroptosis. Specifically, TGFβ1 mitigated neuroinflammation by downregulating inflammasome components such as NLRP3 and IL1β (35). The transcriptomic analysis also showed a reduction in the gene levels of *Nfkbia* and *Nfkbib* among others (**Figure 1E**). This inhibition likely occurs through TGFβ1 dependent signaling, suggesting that TGFβ1 attenuates a broad range of microglial proinflammatory pathways and one possible mechanism is by antagonizing NF-κB-driven transcription of inflammasome components. A similar effect of TGFβ1 on NF-κB signaling was shown by Xie Y et al. (35) in pMG. Their work has shown a notable increase in the translocation of NF-κB into the nuclei of microglial cells upon LPS stimulation. However, treatment with TGFβ1 effectively reversed this activation, leading to a reduced nuclear translocation of NF-κB. Moreover, TGFβ can inhibit LPS induced NF-κB signatures via canonical SMAD and/or non-canonical pathways. In macrophages, downregulation of LPS-induced proinflammatory transcription factors such as *Ap1*, *Atf3* and *Nf-kB* was shown to be mediated by activation of canonical TGFβ signalling component SMAD3. Moreover, TGFβ failed to suppress LPS-induced expression of *Tnfa* and *Il6* in *Smad3-*deficient macrophages, which points to the important role of this transcription factor in TGFβ mediated anti-inflammatory effects (36). In microglia cells, TGFβ1 was shown to interfere with LPS induced changes by targeting MAPK- NF-κB axis (15). Overall, the current findings, combined with previously known actions of TGFβ1, highlight its potential as a therapeutic option in managing neurodegenerative conditions and underlying neuroinflammation.

Our data also demonstrated that BV2 and pMG cells exhibit differences in the activation of inflammasome components upon LPS exposure. While TGFβ1 showed consistent suppression of LPS-induced inflammasome-related genes and proteins in both cell types, pMG showed a markedly pronounced inflammatory response to LPS stimulation compared to BV2 cells. For instance, pMG displayed robust IL1β secretion and a detectable mature IL1β band at 17 kDa (**Figure 4E**) when compared to BV2 cells, suggesting a possible difference in the reactivity of these cell types in response to LPS. These findings align with reports that pMG and BV2 cells display transcriptional differences while also sharing some overlapping features (37, 38). Moreover, it has been reported that these two cell types show differences in the expression of microglia markers and signaling plasticity, especially TGFβ signaling, which was shown to be reduced in BV2 cells when compared to pMG (38, 39). Additionally, inflammasome components revealed temporal differences between BV2 cells and pMG. In BV2 cells, TGFβ1 effectively suppressed LPS-induced upregulation of *Nlrp3, Casp1, Il1b, and Il18* at 6 hours (**Figures 2A, C, E, G**). However, the inhibitory effects diminished by 12 hours, remaining significant only for *Nlrp3* (**Figure 2B**). At the protein level, TGFβ1 reduced NLRP3 (**Figures 3A, B**) and IL1β (**Figures 3E, F**) at both time points but required 12 hours to significantly suppress CASP1 (**Figures 3C, D**). In contrast, pMG exhibited stronger, faster responses as mentioned above. LPS triggered robust upregulation of inflammasome genes within 6 hours, all of which were potently inhibited by TGFβ1 at this early time point (**Figure 4**). Apart from the differences in the inflammasome components between different cell types shown in the current study, previous studies have shown differences in the expression of NLRP3 and IL1β between different regions of the brain and between male and female mice (40). These findings emphasize the importance of validating the results from immortalized cell lines in primary cells, particularly when modelling neuroinflammatory diseases where microglial hyperactivation is pathological and also the need to take the region and sex-specific differences into account.

It is well established that inflammasome activation occurs through a two-step process, first priming via transcriptional upregulation which is then followed by assembly and activation (41). Therefore, we next examined whether TGFβ1 could also suppress inflammasome assembly and subsequent IL1β secretion. To this end, we used Nigericin, a well-established activator of the NLRP3 inflammasome (42). Nigericin triggered a substantial IL1β release from LPS-primed pMG. Similar results were previously reported in LPS-treated murine and human primary microglia in the presence of Nigericin (43, 44). However, TGFβ1 effectively reduced the Nigericin-triggered release of both pro-IL1β (31 kDa) and mature IL1β (17 kDa) (**Figures 5A, B**). These findings were further corroborated by ELISA which showed an approximately 70% reduction in secreted IL1β in the presence of TGFβ1. This effect can likely be attributed to diminished pro-IL1β pools due to TGFβ1’s transcriptional repression, rather than direct interference with NLRP3 oligomerization. This distinction is crucial as it suggests that TGFβ1 inhibits excessive inflammasome activation by reducing LPS-induced priming at an early stage rather than interfering with an already established inflammasome assembly. Interestingly, previous studies have shown beneficial effects upon inhibition of NLRP3 using Nigericin inhibitors such as MCC950/CRID3. In a mouse model of chronic unpredictable mild stress (CUMS), inhibition of NLRP3 inflammasome was shown to reverse the microglial reactivity and morphology via inhibiting NLRP3-Caspase1-IL1β signaling (45). Furthermore, the MCC950 application was shown to attenuate the Aβ+LPS-induced IL1β in pMG and also improved the Aβ-clearance in the APP/PS1 mouse model, indicating the protective outcomes upon inflammasome inhibition (46). Similar effects were also shown on other types of immune cells such as macrophages previously. In LPS-primed bone marrow-derived macrophages (BMMs), MCC950 was shown to significantly suppress Nigericin-induced IL1β secretion and cell death (47). Therefore, in the future, it could be interesting to check if MCC950 accomplishes some of these tasks by activating TGFβ signaling.

**Figure 7:**
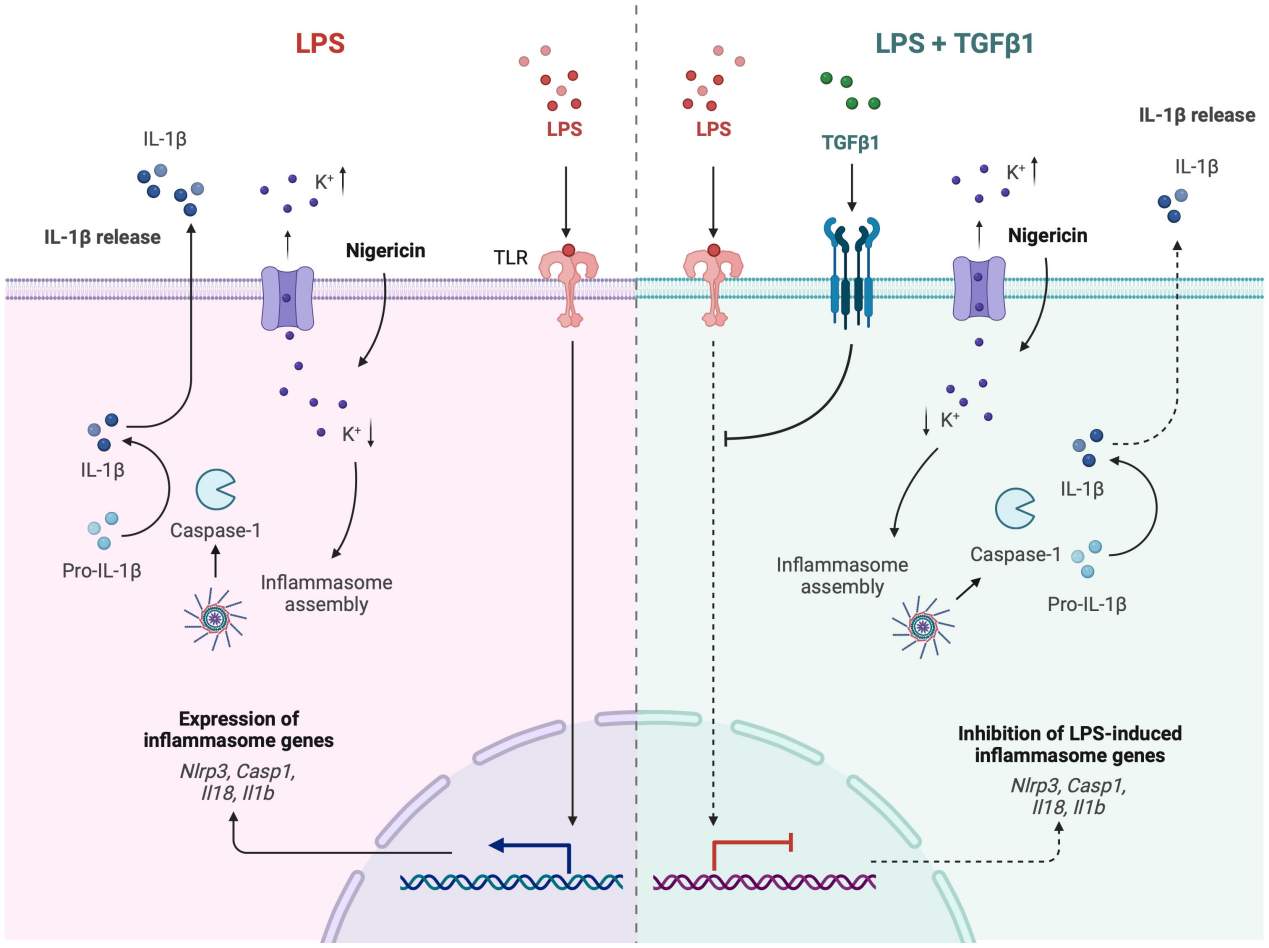
Schematic summary of TGFβ1-mediated regulation of expression of inflammasome genes and activation of the NLRP3 inflammasome in microglia. Created with BioRender.com.

Taken together, our study provides evidence that TGFβ1 serves as a negative regulator of inflammasome activation in microglia by inhibiting the LPS-induced priming step, as shown by of *Nlrp3*, *Casp1*, *Il18*, and *Il1b* suppression at both transcriptional and protein levels. TGFβ1 was also shown to effectively reduce IL1β secretion following inflammasome activation (**Figure 7**). These effects can potentially contribute to the broader protective role of TGFβ1 in maintaining the CNS homeostasis and positions it as a promising target for modulating neuroinflammation in neurodegenerative, acute, and/or chronic brain conditions. Using microglia-specific *Tgfbr2*-deficient mice, we were able to demonstrate that microglial TGFβ signalling is essential to control microglial priming and, thus, expression of inflammasome genes *in vivo*. Future studies should be directed at understanding the status of NLRP3 inflammasome in glial cells beyond microglia, for instance, astrocytes, under homeostatic as well as pathological conditions. Furthermore, utilizing the conditional microglia-specific mutant mice with TGFβ1 signaling deficiency can reveal the changes associated with disease-relevant inflammasome activation. Moreover, the findings from murine microglia could be extrapolated to human cells using inducible microglia models which could yield valuable information regarding the role of TGFβ signaling in regulating inflammasome activation in human diseases.

Additionally, while our findings underscore a regulatory role for TGFβ1 in inflammasome activity, it is important to acknowledge that some recent preclinical studies suggest that inflammasome activation may not be essential for conditions such as Aβ-induced neuropathology (48). This could indicate that inflammasome involvement is more refined than previously thought. These discrepancies also underscore the complexity of neuroinflammation associated with neurodegenerative conditions and the pressing need for further research to fully elucidate the contribution of specific inflammatory pathways to disease progression.

## Data Availability

The cDNA microarray datasets of control and TGFβ1-treated primary microglia cells were generated for a previously published study (16) and are accessible through GEO Series accession number GSE115652.
